# Role of Native Arbuscular Mycorrhizal Fungi in Modulating Nutrient Subcellular Distribution in Wheat Grown in Mn-Toxic Soil

**DOI:** 10.3390/jox15030070

**Published:** 2025-05-11

**Authors:** Jorge Miguel Silva Faria, Ana Paula Pinto, Pedro Barrulas, Isabel Brito, Dora M. Teixeira

**Affiliations:** 1 INIAV, I.P., National Institute for Agrarian and Veterinary Research, Quinta do Marquês, 2780-159 Oeiras, Portugal; 2 GREEN-IT Bioresources for Sustainability, Instituto de Tecnologia Química e Biológica, Universidade Nova de Lisboa (ITQB NOVA), Av. da República, 2780-157 Oeiras, Portugal; 3 Science and Technology School, Évora University, Rua Romão Ramalho nº 59, 7000-671 Évora, Portugal; app@uevora.pt (A.P.P.); pbarrulas@uevora.pt (P.B.); ibrito@uevora.pt (I.B.); dmt@uevora.pt (D.M.T.); 4 MED, Mediterranean Institute for Agriculture, Environment and Development & CHANGE, Global Change and Sustainability Institute, Institute for Advanced Studies and Research, Évora University, Pólo da Mitra, Ap. 94, 7006-554 Évora, Portugal; 5 HERCULES Laboratory & IN2PAST—Associate Laboratory for Research and Innovation in Heritage, Arts, Sustainability and Territory, Évora University, Largo Marquês de Marialva 8, 7000-809 Évora, Portugal

**Keywords:** cell wall immobilization, extraradical mycelium, manganese toxicity, mycorrhiza, nutrient partitioning, wheat

## Abstract

Heavy metal toxicity leads to impaired crop growth and reduced crop yields and product quality by disrupting plant nutrient uptake, inhibiting development, inducing oxidative stress, and causing cellular toxicity. Arbuscular mycorrhizal fungi (AMF) can play a crucial role in crops’ adaptation to manganese (Mn) toxicity by regulating nutrient uptake and altering subcellular compartmentalization. The present study examines the influence of intact extraradical mycelia (ERMs) from native AMF on wheat (*Triticum aestivum*) grown in Mn-toxic soil, with a focus on the tissue-specific and subcellular Ca, Mg, P, and Mn distribution. Wheat cultivated in soil pre-colonized using an intact ERM associated with *Lolium rigidum* or *Ornithopus compressus* exhibited enhanced growth and improved P contents. During the first week of growth, the Mn concentrations increased in the wheat’s roots and shoots, but Mn was subsequently reduced and sequestered within the cell wall. In contrast, in the absence of an intact ERM, the Mn accumulation in wheat followed an apparent continuous time-course pattern. AMF-mediated cell wall sequestration seems to contribute to Mn detoxification by limiting excessive cytoplasmic accumulation. Furthermore, AMF-driven changes in the element distribution suggest a dynamic response, wherein an early-stage nutrient uptake transitions into a long-term protective mechanism. These findings highlight the potential of AMF in mitigating Mn stress in crops, providing insights for sustainable agriculture and soil remediation strategies.

## 1. Introduction

Heavy metals (HMs) are elements with a high atomic weight and density, typically exceeding 5 g/cm^3^. Their levels in the soil at a toxic concentration can substantially impact crop production by reducing the diversity of beneficial microbiota and microfauna, as well as inhibiting plant growth and development [1,2]. Elevated HM levels often come from anthropogenic activities, including industrial processes, coal conversion, and waste incineration. However, natural sources of HMs can also have substantial and widespread effects on soil health and plant ecosystems [3]. Under natural conditions, elements like aluminum (Al), manganese (Mn), and iron (Fe) can reach toxic levels in acidic soils, while important macronutrients become less available [4]. Soil acidification is a natural process that can become aggravated by agricultural practices through an increase in the concentration of hydrogen ions (H^+^) in the soil, primarily after the excessive use of fertilizers and inefficient nitrogen management [5]. Ammonium-based fertilizers release nitrogen, which is quickly converted into nitrate (NO_3_^−^) and H^+^ in the soil. When plants fail to uptake nitrate, it leaches away, leaving behind H^+^, which contributes to acidification. Manganese toxicity, driven by soil acidity, poses a significant challenge in many agricultural soils worldwide [6]. Under Mn toxicity, crops exhibit stunted growth and reduced productivity, accompanied by symptoms such as chlorosis in older leaves, necrotic spots, white flecking, purpling, and leaf tip burn, progressing from older to younger leaves [7,8]. Elevated Mn levels in the soil solution can disrupt the uptake of other essential nutrients, such as magnesium (Mg) and calcium (Ca), through competition for shared transport pathways [9,10,11]. Diagnostics is often challenging, as the symptoms of Mn toxicity are sometimes mistaken for Fe deficiency [12].

Acidic soils with high levels of bioavailable Mn are also a driver of the adaptability of native microbiota. Under these challenging conditions, soil microorganisms have developed strategies for enduring environmental extremes [13,14]. Notably, some mycotrophic plants can thrive in Mn-toxic soils, aided by arbuscular mycorrhizal fungi (AMF), especially if they are promptly colonized by an intact extraradical mycelium (ERM) as a preferential source of inoculum [15]. Arbuscular mycorrhizal fungi establish arbuscules within the root cells and form a large ERM extending outside the roots, which can cover over 100 m of hyphae per cubic centimeter of soil [16]. Besides contributing to the stability of the soil structure [17], the ERM plays a crucial role in mobilizing water and nutrients, primarily P, while the arbuscule facilitates their exchange with photosynthetic products from the host—accounting for between 10 and 30% of the hosts’ photosynthetic output [18]. Under HM toxicity, AMF are known to benefit plants by improving the soil’s physicochemical properties and filtering xenobiotics within their mycelium, effectively acting as a protective physical barrier for the mycorrhizal plant (e.g., by chelating HMs in the cell wall or to the glomalin produced by the AMF and released into the soil) [19,20]. Additionally, AMF can immobilize HMs at the plants’ roots. More importantly, they enhance plants’ development and efficiency by influencing the host’s biochemistry, optimizing redox balance, and facilitating intracellular compartmentalization of HMs [21], as reviewed in detail by [20,22]. Wheat grown in Mn-toxic soil attempts to limit its Mn uptake at the roots, albeit inefficiently, and sequesters excess Mn into the vacuoles of its shoot cells [23]. However, when a highly mycotrophic plant, such as *Lolium rigidum* L. (LOL) or *Ornithopus compressus* L. (ORN), is grown beforehand and establishes its characteristic AMF consortium in the soil, subsequent plants appear to tolerate the toxicity caused by excess Mn better [24]. Under these conditions, an undisturbed ERM formed through previous symbiosis with LOL or ORN will colonize the wheat roots more rapidly than in its absence, conferring earlier protective benefits by stimulating growth, reducing Mn uptake, and altering the subcellular ratios of Mn and other nutrients [21,25].

The mechanisms underlying the protection conferred by intact ERMs from native AMF in wheat’s growth in Mn-toxic soils remain unclear. However, earlier studies have identified differential gene expression in wheat roots colonized by ERMs previously developed through symbiotic LOL-AMF or ORN-AMF combinations. Through LOL-AMF symbiosis, wheat genes associated with oxidative stress response, disease resistance, and metal ion binding were predominantly upregulated. Conversely, in wheat roots grown after colonization by the ORN-AMF consortium, the upregulated genes were primarily linked to cellular division and growth, with minimal association with stress responses [26].

The present study aims to contribute to uncovering these biochemical mechanisms by detailing the compartmentalization of the nutrients Mg, Ca, P, and toxic Mn at the tissue and subcellular levels in the shoots and roots of wheat grown after LOL-AMF or ORN-AMF symbiosis weekly over 3 weeks. This was performed to elucidate the dynamics of elemental displacement following the colonization of intact ERMs with different communities of beneficial native AMF, depending on the plant grown before wheat. The outcomes of this study elucidated the mechanisms through which an intact ERM from native arbuscular mycorrhizal fungi (AMF) enhances wheat growth in Mn-toxic soil. Primarily, the ERM appears to facilitate Mn uptake during the early stages of colonization, likely triggering an early tolerance response that promotes enhanced growth at later developmental stages. To our knowledge, this is the first report of this mechanism in this system.

## 2. Materials and Methods

### 2.1. The Soil’s Characteristics, the Plant Material, and the Experimental Design

The soil used was a granitic Eutric Cambisol with Mn toxicity related to its granitic origin, collected from the top 20 cm layer at the Mitra Farm, the University of Évora, Alentejo, Portugal (38°32′ N; 08°00′ W). This sandy loam soil has previously been chemically analyzed [27]. The key nutrient concentrations were 67 mg/kg of K, determined using the Egner–Riehm method combined with flame atomic emission spectroscopy; 112 mg/kg of Mg, obtained using 1 M ammonium acetate at a pH of 7 and analyzed through atomic absorption spectroscopy; 41 mg/kg of Mn, assessed through the Lakanen method using flame atomic absorption; 26 mg/kg of P, quantified through UV–Vis molecular absorption spectrophotometry after Egner–Riehm extraction; and 0.4 mg/kg of N-NO_3_. The soil organic matter (SOM) was previously determined to be 11 g/kg (chromic acid wet oxidation), and the cation exchange capacity (CEC) was 4.5 centimoles of charge per kg [cmol(+)/kg], with a base saturation of 60% and a pH of 5.6 at a 1:2.5 soil-to-water ratio (*w*/*v*) [27]. The soil solution Mg, Ca, P, and Mn were determined to be 22.4 ± 1.3, 0.02 ± 0.00, 50.2 ± 3.0, and 5.7 ± 0.7 mg/kg of soil dry weight (DW), respectively, through ICP-MS according to [4], after soil solution isolation by centrifuging ca. 40 g of soil at 4 °C for 60 min at 2500× *g* using 50 mL tubes equipped with 0.45 µm polyethersulfone membrane filters. The abundance of AMF in this soil was previously quantified at 180 viable propagules per g DW of soil, and the AMF diversity was also previously profiled [27,28].

To prime the Mn-toxic soil with ERMs from beneficial AMF, the collected soil was used to grow each of the mycotrophic species. Thus, dark plastic pots with a capacity of 8 L were filled with the homogenized soil, moistened to approximately 70% of its maximum water holding capacity (by weight), and maintained under these conditions in a greenhouse for one week to allow for soil stabilization. Throughout this period, the daily minimum and maximum air temperatures were recorded, with the temperature regulation system set not to exceed 30 °C. Afterwards, five ORN or LOL seedlings, germinated in hydrated filter paper, were selected at a similar development stage and planted at equally distanced positions in each of twelve replicate pots for each treatment [24]. As a control, five *Silene gallica* L. (SIL) seedlings, a non-mycotrophic plant that does not develop ERMs, were also planted as described above. The AMF developer species were selected based on prior studies demonstrating the beneficial effects of their specific AMF consortia on wheat growth, including their association with reduced internal Mn levels in wheat leaves [27,28]. The pots were kept within the conditions described above for 7 weeks to allow the mycotrophic plants to develop naturally and establish symbiotic relationships with their preferred AMF symbionts. Any germinated weeds were excised to avoid interference from different hosts in the formation of the AMF consortium. Following their growth period, the mycotrophic plants were removed through cutting, and six uniformly spaced seedlings of wheat (*Triticum aestivum* L., cultivar Ardila) were sown in each pot. The pots were then arranged in a completely randomized design within the greenhouse. After 1, 2, and 3 weeks, the wheat plants were retrieved from four replicate pots at each time point, and the roots and shoots were weighed separately. Three plants per pot were used to determine the tissue DW after drying at 60 °C for 3 days. For the remainder, the tissues were immediately frozen in liquid nitrogen and stored at −80 °C until analysis. In previous studies, for wheat plants grown in soil from LOL or ORN, the mycorrhizal colonization was previously confirmed to be 51% and 74% of the root length, respectively, while no colonization was reported for SIL [15].

### 2.2. Tissue and Subcellular Element Partitioning

For the shoot or root tissues, two fractions were isolated: a fraction enriched in cell walls and a fraction enriched in cellular contents [27]. Thus, frozen wheat shoots were pulverized using liquid nitrogen and then homogenized in buffer composed of 250 mM sucrose, 1.0 mM dithioerythritol, and 50 mM Tris–HCl at a pH of 7.5, using a ratio of 200 mg of plant tissue per 5 mL of buffer. Afterwards, this suspension was centrifuged at 2500× *g* at 4 °C for 15 min, and the supernatant was isolated from the pellet. The supernatant was composed of organelle components (i.e., chloroplasts, mitochondria), cytoplasm, and vacuole contents (consisting of metal-binding compounds such as phytochelatins and metallothioneins, as well as antioxidant enzymes), and the pellet corresponded to the cell wall fraction, mainly composed of cell walls, cellular debris, and any metal granules. Both fractions were frozen and kept at −80 °C until analysis.

### 2.3. Microwave-Assisted Acidic Digestion of the Wheat Tissues and Subcellular Fractions

Before the elemental analysis, the wheat roots, shoots, and respective fractions were digested in acidic conditions to obtain elemental homogenous solutions. The ground shoot and root samples (50 mg), as well as the respective subcellular fractions, were first lyophilized in a Telstar ^®^ LyoQuest lyophilizer for 3 days in Teflon vessels. Next, 3 mL of HNO_3_ (Suprapur, 67–69%, Fisher Chemicals, Hampton, NH, USA) and 2 mL of HCl (Trace Metal Grade, 37%, Fisher Chemicals) were added to sealed Teflon vessels and allowed to stand overnight at an ambient temperature. The mixtures were then subjected to microwave-assisted acid digestion, carried out at 240 °C for 40 min, followed by a 15 min cooling phase, using a Mars 6 microwave digestion system (CEM, Matthews, NC, USA). The accuracy of the method and the detection limits were assessed by including a digestion blank and a certified reference material (NIST SRM 1573a, Tomato leaves, Gaithersburg, MD, USA) in each digestion batch [23]. The digested plant solutions and soil solutions were filtered using 0.45 µm PTFE membrane filters, and ultrapure water was added to adjust the volume to 20 mL. These prepared solutions were then immediately analyzed via ICP-MS.

### 2.4. Quantitative Analysis of Mg, Ca, P, and Mn

The diluted samples (40- and 1000-fold) were analyzed in an 8800 Triple Quadrupole inductively coupled plasma mass spectrometer (Agilent, Santa Clara, CA, USA) equipped with a Micromist nebulizer [29]. An Agilent ICP–MS tuning solution of 2% HNO_3_ containing 10 μg/L each of Ce, Co, Li, Tl, and Y (Agilent Technologies, Palo Alto, CA, USA) was used for instrument calibration, sensitivity optimization, and interference reduction. External calibration was performed using the multi-element certificate standard solution ICP–MS-68B-A (100 mg/L) from High-Purity Standards (Charleston, SC, USA). Matrix effects and instrumental drifts were corrected based on the internal standards ruthenium (Ru), rhodium (Rh), and iridium (Ir). The collision/reaction cell was set to “no-gas mode” for the quantification of Mn and Mg, “O_2_ mode” for the quantification of P, and “NH_3_ mode” for the quantification of Ca. The plasma gas flow rate was 15 mL/min, and the collision and reaction gas flow rates for He, O_2_, and NH_3_ were 4.0 mL/min, 0.5 mL/min, and 1.5 mL/min, respectively. The analyses were optimized at a 1550 W forward power and a 1.1 L/min Ar carrier gas flow, with no dilution or makeup gas. The sampling depth (10 mm) and lens parameters were optimized for the highest signal and optimum peak shape while maintaining low oxides and doubly charged species. The MS/MS scan type was used in all operation modes. The element levels were expressed per root or shoot DW.

### 2.5. The Data Treatment and Statistical Analysis

Statistical processing was performed using version 26 of SPSS Statistics software (IBM, New York, NY, USA). The statistical significance of the data was determined using a one-way ANOVA, and individual means were compared using Tukey’s post hoc test with *p* < 0.05 (Shapiro–Wilk test ensured the data normality, and the Browns–Forsythe test was used for homoscedasticity). The results were presented as the average and standard error values of four replicates.

## 3. Results

### 3.1. Wheat Growth

**Figure 1:**
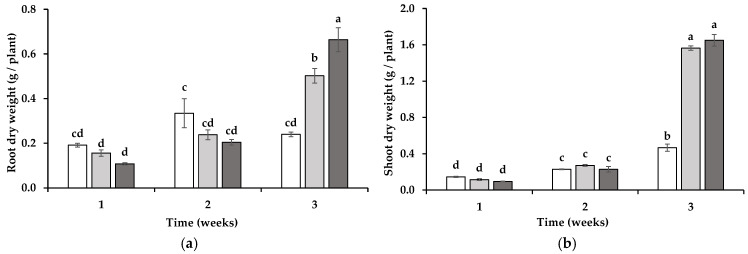
Growth assessed through root (**a**) and shoot (**b**) dry weight of wheat grown for 1, 2, and 3 weeks in soil in which non-mycotrophic *Silene gallica* (white columns), mycotrophic *Lolium rigidum* (light gray columns), or *Ornithopus compressus* (dark gray columns) had previously been grown. Different letters indicate statistically significant differences (*p* < 0.05) on the basis of Tukey’s test.

The growth pattern of the wheat seedlings developed in Mn-toxic soil varied depending on whether the soil had previously been cultivated with a mycotrophic or non-mycotrophic plant species. During the first and second weeks of development, no significant differences were observed in the root biomass between the treatment groups. However, by the third week, the root DW was highest in the wheat plants grown in soil previously cultivated with the mycotrophic ORN, reaching 0.66 ± 0.05 g DW/plant. The roots of the wheat grown in soil that had previously harbored the mycotrophic LOL also exhibited a substantial increase in their DW, at 0.50 ± 0.03 g DW/plant, which was significantly lower (*p* < 0.05) than the dry weight of the wheat from the ORN-cultivated soil but more than twice the root biomass observed in the wheat grown in soil previously cultivated with the non-mycotrophic SIL, at 0.24 ± 0.01 g DW/plant (Figure 1a).

A similar trend was observed in the shoot biomass of wheat. By the third week, the shoot DW of the wheat grown in soil previously cultivated with ORN was 1.65 ± 0.06 g DW/plant, slightly higher than that of wheat from LOL-treated soil (1.56 ± 0.03 g DW/plant) (not statistically significant). Both values were markedly greater than the shoot biomass of the wheat grown in soil previously cultivated with SIL, which was limited to 0.47 ± 0.04 g DW per plant (Figure 1b).

Regarding wheat growth, the influence of an intact ERM becomes apparent only after three weeks of symbiosis. However, it is possible that chemical or biochemical changes occur at earlier stages of colonization.

### 3.2. Tissue Element Levels

**Figure 2:**
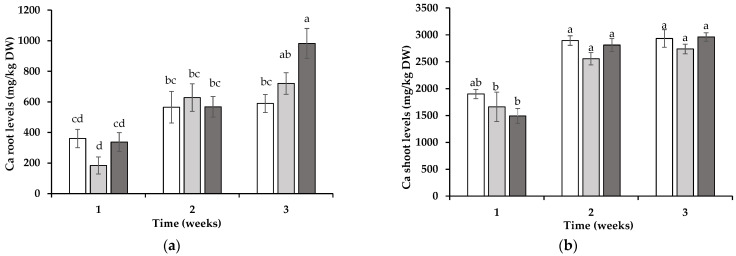

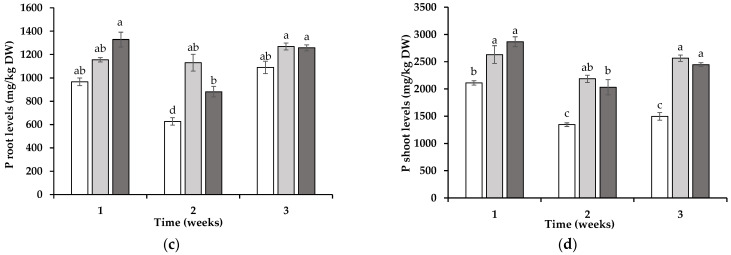
Calcium (Ca) (**a**,**b**) and phosphorous (P) (**c**,**d**) levels in roots (**a**,**c**) and shoots (**b**,**d**) of wheat planted in soil into which non-mycotrophic *Silene gallica* (white columns), mycotrophic *Lolium rigidum* (light gray columns), or *Ornithopus compressus* (dark gray columns) had previously been grown, as monitored for 1, 2, and 3 weeks. Different letters indicate statistically significant differences (*p* < 0.05) on the basis of Tukey’s test.

The concentrations of Ca, P, Mg, and Mn were monitored in the roots and shoots of wheat over a 3-week period under the three distinct soil conditions. The Ca concentrations in the roots of wheat grown for 1 or 2 weeks did not exhibit substantial differences across treatments. However, by the third week, the Ca levels in the roots were significantly higher (*p* < 0.05) in the wheat grown in soil into which ORN had previously been grown (982 ± 98 mg/kg DW) compared to those in the wheat roots from the soil previously planted with SIL (589 ± 59 mg/kg DW) (Figure 2a). The Ca levels in the roots of wheat grown in soil previously planted with LOL (720 ± 44 mg/kg DW) did not differ significantly from the levels in those grown in the soils that previously harbored ORN or SIL. In the wheat shoots, the Ca concentrations remained consistent across treatments at each time point, with no statistically significant differences detected (Figure 2b).

For wheat root P content, statistically significant differences were observed only during the second week. The wheat plants grown following the development of LOL or ORN exhibited higher P concentrations in their roots compared to these values in those grown after SIL (Figure 2c). In the wheat shoots, the influence of AMF symbiosis was evident as early as the first week, with the P concentrations in the shoots being significantly higher (*p* < 0.05) in wheat grown after LOL (2630 ± 164 mg/kg DW) or ORN (2867 ± 89 mg/kg DW) relative to these values in the wheat grown after SIL (2111 ± 40 mg/kg DW) (Figure 2d). These differences became more pronounced over time, with the P levels in the wheat shoots grown in Mn-toxic soil after LOL and ORN treatment increasing 1.6- and 1.5-fold, respectively, during the second week and 1.7- and 1.6-fold, respectively, during the third week.

**Figure 3:**
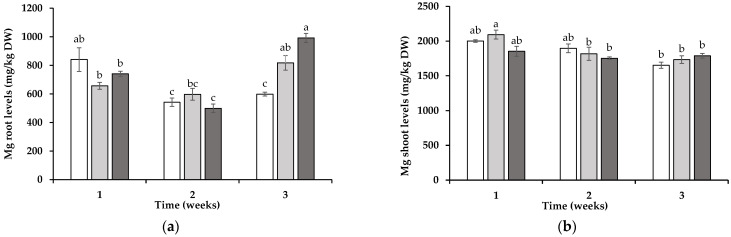

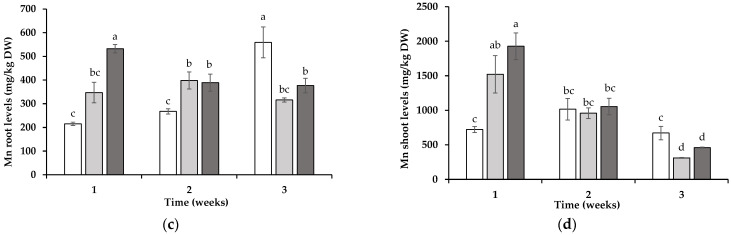
Magnesium (Mg) (**a**,**b**) and manganese (Mn) (**c**,**d**) levels in roots (**a**,**c**) and shoots (**b**,**d**) of wheat planted in soil in which non-mycotrophic *Silene gallica* (white columns), mycotrophic *Lolium rigidum* (light gray columns), or *Ornithopus compressus* (dark gray columns) had previously been grown as monitored for 1, 2, and 3 weeks. Different letters indicate statistically significant differences (*p* < 0.05) on the basis of Tukey’s test.

The magnesium concentrations in the wheat roots showed no statistically significant differences during the 1st and 2nd weeks. However, by the 3rd week, the Mg levels were significantly higher (*p* < 0.05) in the roots of the wheat grown in soil previously cultivated with ORN (991 ± 32 mg/kg DW) compared to the wheat grown in soil previously cultivated with SIL (598 ± 13 mg/kg DW) (Figure 3a). The magnesium concentrations in the roots of wheat grown in soil previously cultivated with LOL (817 ± 51 mg/kg DW) were not significantly different from the concentrations in that grown in soil previously cultivated with either ORN or SIL. In the wheat shoots, the Mg levels remained relatively consistent across the treatments at different time points, with no statistically significant differences being observed (Figure 3b).

The manganese concentrations exhibited significant variations across treatments and time points. By the end of the first week, the Mn levels in the wheat roots were highest in the plants grown in soil previously cultivated with ORN (532 ± 7 mg/kg DW), followed by those grown after LOL (347 ± 43 mg/kg DW), and were lowest in the roots of the wheat grown after SIL (215 ± 7 mg/kg DW) (Figure 3c). During the second week, these differences diminished; however, the root Mn concentrations of wheat grown after LOL or ORN remained approximately 1.5-fold higher than those in the wheat grown after SIL. By the third week, this trend was reversed, with the roots from the SIL treatment exhibiting significantly higher Mn levels (559 ± 64 mg/kg DW) compared to those in the roots from the LOL (315 ± 9 mg/kg DW) or ORN (376 ± 30 mg/kg DW) treatments (*p* < 0.05) (Figure 3c).

In the wheat shoots, a similar progressive pattern was observed. In the first week, the shoot Mn levels were higher in the wheat grown after ORN (1927 ± 193 mg/kg DW) and LOL (1521 ± 270 mg/kg DW) compared to that grown after SIL (720 ± 44 mg/kg DW) (Figure 3d). By the second week, no statistically significant differences in the shoot Mn levels were detected among treatments. However, in the third week, the Mn concentrations in the shoots of the wheat grown after SIL remained high (671 ± 97 mg/kg DW), while the levels in the shoots from the LOL (311 ± 6 mg/kg DW) and ORN (459 ± 8 mg/kg DW) treatments markedly declined (Figure 3d).

### 3.3. Tissue Subcellular Redistribution

**Figure 4:**
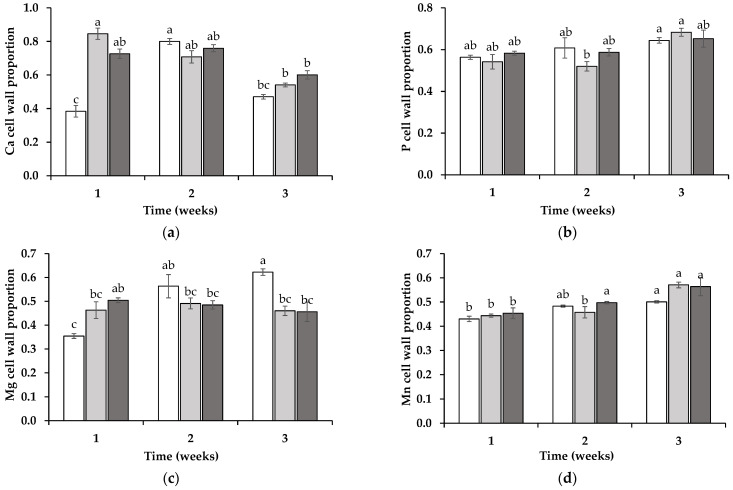
The ratio of calcium (Ca) (**a**), phosphorus (P) (**b**), magnesium (Mg) (**c**), and manganese (Mn) (**d**) in the roots of wheat grown in soil in which non-mycotrophic *Silene gallica* (white columns), mycotrophic *Lolium rigidum* (light gray columns), or *Ornithopus compressus* (dark gray columns) had previously been grown as assessed for 1, 2, and 3 weeks. Different letters indicate statistically significant differences (*p* < 0.05) on the basis of Tukey’s test.

The subcellular redistribution of Ca, P, Mg, and Mn was monitored in the roots and shoots of the wheat over three weeks of development in soil containing intact ERMs of ORN or LOL or in soil without the prior presence of a mycotrophic plant. For root Ca, statistically significant differences between treatments were observed only during the first week, where the wheat roots grown in soil from the LOL (85 ± 3%) and ORN (73 ± 3%) treatments had a higher proportion of Ca in the cell wall compartment compared to that in the wheat grown in the SIL-treated soil (38 ± 4%) (*p* < 0.05) (Figure 4a). For P, no statistically significant differences were detected, with the proportions in the cell wall fraction ranging from 52 ± 3% to 64 ± 1%. For Mg, during the first week, the roots grown in the LOL- (46 ± 3%) and ORN-treated (50 ± 1%) soil showed higher proportions of Mg in the cell wall compartment compared to that in those from the SIL treatment (35 ± 1%) (Figure 4b). By the third week, this pattern had been reversed, with a higher proportion of Mg in the cell walls of the roots from the SIL treatment (62 ± 1%) than that in those from the LOL (46 ± 2%) or ORN (46 ± 4%) treatments (Figure 4c). For Mn, no statistically significant differences were observed between treatments, but the Mn levels in the root cell walls increased over time, from between 43 ± 1% and 45 ± 2% in the first week to between 50 ± 1% and 57 ± 1% by the third week (Figure 4d).

**Figure 5:**
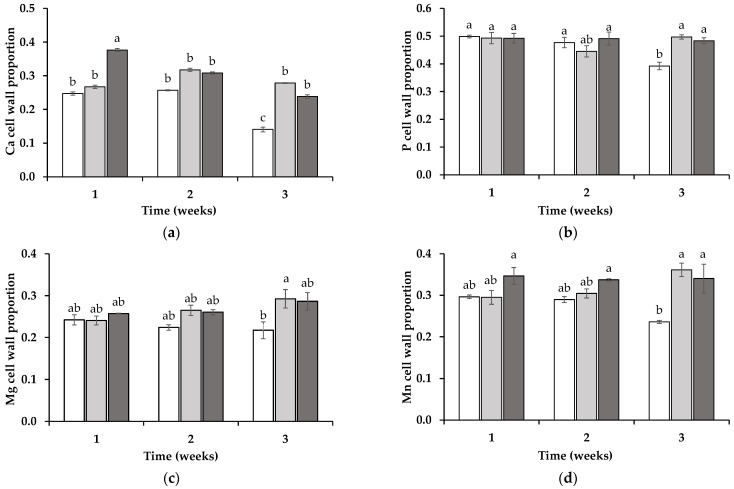
Ratio of calcium (Ca) (**a**), phosphorus (P) (**b**), magnesium (Mg) (**c**), and manganese (Mn) (**d**) in the shoots of wheat grown in soil in which non-mycotrophic *Silene gallica* (white columns), mycotrophic *Lolium rigidum* (light gray columns), or *Ornithopus compressus* (dark gray columns) had previously grown, as assessed for 1, 2, and 3 weeks. Different letters indicate statistically significant differences (*p* < 0.05) on the basis of Tukey’s test.

In the wheat shoots, the subcellular element redistribution differed notably from that observed in the roots. For Ca, during the first week, the wheat plants grown in the soil from the SIL (25 ± 1%) and LOL (27 ± 1%) treatments exhibited significantly lower proportions of Ca in the cell wall compartment compared to this property in those grown in soil from the ORN treatment (38 ± 1%) (*p* < 0.05) (Figure 5a). By the second week, no significant differences were observed between the treatments. However, by the third week, wheat grown in soil from the SIL treatment (14 ± 1%) showed a significantly lower proportion of Ca in its cell walls compared to that in the wheat grown in the LOL (28 ± 1%) or ORN (24 ± 1%) soil treatments (*p* < 0.05). For P, statistically significant differences were observed only in the third week, with wheat grown in the soil from the LOL (50 ± 1%) or ORN (48 ± 1%) treatments displaying significantly higher proportions of P in its cell walls compared to that in the wheat grown in the soil from the SIL treatment (39 ± 1%) (Figure 5b). A similar trend was observed for Mg and Mn in the third week, with wheat grown in soil from the LOL (29 ± 2% and 36 ± 3%, respectively) and ORN (29 ± 2% and 34 ± 3%, respectively) treatments showing significantly higher proportions of Mg or Mn in the cell wall compartment (*p* < 0.05) compared to that grown in the SIL-treated soil (22 ± 2% and 24 ± 1%, respectively) (Figure 5c,d).

## 4. Discussion

The benefits of AMF symbiosis for crop growth and productivity are well documented in the scientific literature, particularly regarding their role in enhancing P uptake and improving plant–water relations under both optimal and stress conditions, as reviewed in [30]. However, our understanding of AMF-mediated nutrient dynamics beyond those for P remains relatively limited. This is particularly evident for other essential nutrients, such as Mg or Ca, which are crucial for plant growth, metabolic regulation, and stress resilience, especially under heavy metal toxicity. This limited understanding arises from the complex and often context-dependent effects of different AMF species on the nutrient uptake, translocation, and distribution within plant tissues. In the present study, the impact of native AMF consortia on wheat’s growth and nutrient compartmentalization under Mn stress was investigated. Wheat grown in soil containing an intact ERM from AMF—previously established through symbiosis with highly mycotrophic plants—exhibited more than double the growth of the wheat grown in soil without a previously developed ERM. This striking improvement in growth highlights the critical role of intact ERMs in mitigating Mn toxicity and supporting nutrient acquisition. However, significant growth was only observed three weeks after planting, indicating that time is required to see the full benefits of AMF symbiosis. Notably, changes in the host plant’s biochemical responses were evident as early as the first week, suggesting that AMF-induced modifications in nutrient dynamics and stress tolerance begin during the early stages of colonization. An increase in the P concentrations in the shoots during the first week suggests the early establishment of AMF symbiosis. This prompt colonization is driven by the nature of the initial inoculum, as a fully developed ERM more efficiently colonizes wheat roots compared to colonization initiated by AMF spores in the soil (i.e., soil from the previously grown non-mycotrophic *S. gallica*) [15]. The immediate establishment of symbiosis seems to greatly enhance the Mn uptake by the roots and its subsequent translocation to the shoots, a response that is particularly pronounced in wheat associated with the ORN AMF. By the second week, the wheat influenced by the ORN AMF begins to exhibit a decline in the Mn concentrations in its roots, potentially signaling a reduction in uptake activity. Conversely, in the wheat grown in soil lacking a pre-established ERM, the Mn concentration continues to rise by the third week. In the presence of an intact ERM formed in association with mycotrophic plants (ORN or LOL), the Mn concentrations decline in both the shoots and roots. This initial surge in Mn uptake may reflect an AMF-associated enhancement in nutrient transport as the AMF hyphae actively mobilize P from the soil to the plant. The increased wheat growth in the soil from mycotrophic plants appears to result primarily from elevated internal P levels and reduced Mn levels, both influenced by early root colonization through the intact pre-formed ERM. Additionally, this condition led to increased root Ca and Mg nutrient levels, further contributing to improved plant health.

The uptake of P and Mn exhibits some interlinkages, with several mechanisms suggesting their coupled dynamics in the soil–plant system. For instance, inositol hexaphosphate (commonly known as phytate), a predominant form of organic P in many soils, has the capacity to form stable complexes with bioavailable Mn [31]. This interaction highlights the role of organic P compounds in indirectly modulating Mn dynamics. Also, an important strategy employed by plants for P acquisition involves the exudation of carboxylates into the rhizosphere. These low-molecular-weight organic acids facilitate the mobilization of P that is bound to Fe and Al hydroxides via ligand exchange reactions, thereby increasing the bioavailability of P for root uptake [32]. However, this strategy also inadvertently mobilizes Mn in the soil, which often results in increased Mn uptake and its subsequent accumulation in plant tissues, particularly in the leaves [33]. Intriguingly, the transport of Mn across plant membranes appears to exhibit low specificity. A significant proportion of the membrane transport proteins responsible for Mn translocation are not exclusively dedicated to Mn. Instead, they also mediate the transport of other divalent cations, including Fe, Zn, Cu, Cd, Ca, Co, and Ni [34]. This lack of specificity is likely driven by the chemical similarities between these cations, particularly in terms of their ionic radii and charge. Consequently, competition among these cations for transport pathways can influence Mn uptake and distribution, further complicating its regulation within plant tissues. Typically, it is believed that Mn and P compete for absorption by plant roots, meaning that the increased absorption of one element results in the reduced uptake of the other. This happens because both elements have similar chemical characteristics, and the transport mechanisms in the roots can be affected by this competition. However, the present study suggests in the presence of a pre-established ERM, this competition process between Mn and P does not occur as expected. In Mn-toxic soil (where the Mn concentrations are very high and can be harmful to the plant), wheat’s uptake of Mn and P seems to be linked. That is, even under conditions of excess Mn, the plant absorbs both elements more effectively with the help of the native AMF, rather than experiencing direct competition.

The competitive interactions among the analyzed elements may partially explain the distinct compartmentalization patterns for Ca, Mg, and Mn observed in the present study. Although the Ca and Mg concentrations remained unchanged during the first week, their subcellular distribution was altered. In the presence of an intact ERM, the Ca and Mg ratios were elevated in the root cell walls, indicating preferential accumulation in this compartment. In wheat shoots, Ca was specifically redirected to the cell wall fraction under the influence of the ORN AMF. By the third week, the Mg in the roots exhibited an inverse trend, with reduced accumulation in the cell wall compared these conditions without an intact ERM. At this stage, the allocation of Ca, P, Mg, and Mn into the cell wall fraction in the shoots increased, driven by the presence of an intact ERM. These findings suggest that Mn is rapidly assimilated within the first week, whereas Mg and Ca are retained in the cell walls. By the third week, these elements are actively transported from the cell wall into the symplast in the roots, likely at the expense of Mn uptake. In the shoots, any excess Mn, Ca, or Mg was sequestered into the cell wall, potentially mitigating Mn toxicity.

When there is excess Mn, it is believed that wheat mitigates its toxicity by sequestering the excess Mn into vacuoles, effectively isolating it from sensitive cellular components and reducing its harmful effects on cellular metabolism [23]. However, the presence of an intact ERM appears to circumvent this sequestration mechanism, favoring Mn’s compartmentalization within the cell wall instead. Cell wall compartmentalization is a crucial mechanism through which plants mitigate Mn toxicity. The cell wall, primarily composed of polysaccharides such as cellulose, hemicellulose, and pectin, along with structural proteins and lignin, can serve as a buffer modulating cation uptake [35]. Negatively charged functional groups, particularly the carboxyl groups of pectin, facilitate cation binding, forming stable complexes that restrict mobility. Pectin, rich in polygalacturonic acid residues, exhibits a high affinity for divalent cations, also contributing to Mn sequestration [36,37]. This apoplastic cation loading maintains elevated concentrations of polyvalent cations near the plasma membrane or along the apoplasmic pathway, which is critical for the uptake of certain macronutrients. A low external pH decreases the charge density of the cell walls so that the effects on cation uptake become more pronounced. Additionally, competition between Mn and other divalent cations, such as Ca and Mg, for binding sites further modulates Mn compartmentalization [36]. In response to Mn stress, plants can also modify the cell wall’s composition to enhance Mn immobilization, including increased pectin synthesis and altered lignin deposition. Lignification of the cell wall reduces Mn mobility, thereby limiting its cytoplasmic entry [8]. Furthermore, enzymatic redox reactions, mediated by peroxidases, oxidize bioactive Mn into insoluble Mn oxides, which are subsequently immobilized in the cell wall [38].

In the present study, the temporal shift in the Mn distribution may also have been tied to the activation of the host plant’s responses mediated by the AMF. Early in the interaction, AMF likely prioritize colonization and nutrient uptake, leading to increased Mn translocation into plant tissues. As the symbiosis develops, stress-alleviating pathways such as antioxidant enzyme activation, redox balance optimization, and selective nutrient transport come into play, reducing the negative impacts of Mn toxicity on wheat growth [24,27,39]. This delayed reduction in Mn levels highlights the dynamic nature of AMF symbiosis. The initial spike in Mn may serve as a short-term adaptation to enhance nutrient acquisition, whereas the subsequent reductions reflect the establishment of long-term protective mechanisms. In previous work, we analyzed the changes in wheat roots’ transcriptome following colonization by these specific AMF consortia. Our findings revealed the upregulation of several transporter genes, including the heavy metal-transporting P1B-ATPase 2, five phosphate transporters—none of which had been previously identified as AMF-inducible—and two ETHYLENE-INSENSITIVE 2 (EIN2) genes from the NRAMP family of metal transporters [26].

Future research should investigate the specific biochemical and molecular changes occurring in wheat during this temporal shift. A deeper understanding of how AMF modulate Mn transporters and detoxification pathways could reveal novel strategies for enhancing plants’ resilience in soils exhibiting Mn toxicity. Additionally, exploring how these dynamics vary across different AMF consortia and host plants may offer insights for optimizing AMF-based biotechnological solutions in sustainable agriculture.

## 5. Conclusions

This study provides evidence for the role of AMF in enhancing wheat’s growth and nutrient dynamics in Mn-toxic soils. The temporal patterns of Mn uptake and detoxification, coupled with the redistribution of essential nutrients, emphasize the multifaceted benefits of AMF symbiosis. These findings pave the way for further exploration of AMF-mediated mechanisms and their application to developing sustainable agricultural practices.

## Data Availability

The raw data supporting the findings of this study are available from the corresponding author (Jorge M. S. Faria) upon reasonable request.
